# RETRACTED: Cheng et al. High PGC-1α Expression as a Poor Prognostic Indicator in Intracranial Glioma. *Biomedicines* 2024, *12*, 979

**DOI:** 10.3390/biomedicines13122899

**Published:** 2025-11-27

**Authors:** Yu-Wen Cheng, Jia-Hau Lee, Chih-Hui Chang, Tzu-Ting Tseng, Chee-Yin Chai, Ann-Shung Lieu, Aij-Lie Kwan

**Affiliations:** 1 Department of Neurosurgery, Kaohsiung Veterans General Hospital, Kaohsiung 807, Taiwan; 2 Graduate Institute of Medicine, College of Medicine, Kaohsiung Medical University, Kaohsiung 807, Taiwan; 3 National Institute of Cancer Research, National Health Research Institutes, Tainan 701, Taiwan; 4 Division of Neurosurgery, Department of Surgery, Kaohsiung Medical University Hospital, Kaohsiung 807, Taiwan; 5 Department of Pathology, Kaohsiung Medical University Hospital, Kaohsiung 807, Taiwan; 6 Department of Pathology, School of Medicine, College of Medicine, Kaohsiung Medical University, Kaohsiung 807, Taiwan; 7 Department of Surgery, School of Medicine, College of Medicine, Kaohsiung Medical University, Kaohsiung 807, Taiwan; 8 Department of Neurosurgery, University of Virginia, Charlottesville, VA 23806, USA

The journal retracts the article “*High PGC-1α Expression as a Poor Prognostic Indicator in Intracranial Glioma*” [1] cited above.

Following publication, concerns were brought to the attention of the publisher regarding a partial overlap of images presented in this article [1].

Adhering to our complaint’s procedure, an investigation was conducted that confirmed a partial duplication within Figure 5, presenting different experimental conditions. While the authors cooperated with the Editorial Office during the investigation, they were unable to satisfactorily explain the similarities within Figure 5, nor provide raw material for the Editorial Board to review.

As a result, the Editorial Board has lost confidence in the reliability of the findings and has decided to retract this publication [1], as per MDPI’s retraction policy (https://www.mdpi.com/ethics#_bookmark30).

This retraction was approved by the Editor-in-Chief of the *Biomedicines* journal.

The authors disagree with this retraction.
